# *N*-(2,4-Dimethyl­phen­yl)-4-methyl­benzamide

**DOI:** 10.1107/S1600536810013413

**Published:** 2010-04-21

**Authors:** Vinola Z. Rodrigues, Miroslav Tokarčík, B. Thimme Gowda, Jozef Kožíšek

**Affiliations:** a Department of Chemistry, Mangalore University, Mangalagangotri 574 199, Mangalore, India; b Faculty of Chemical and Food Technology, Slovak Technical University, Radlinského 9, SK-812 37 Bratislava, Slovak Republic

## Abstract

In the mol­ecule of the title compound, C_16_H_17_NO, the N—H and C=O bonds are *anti* to each other and the two benzene rings form a dihedral angle of 75.8 (1)°. The amide group is twisted by 28.1 (3) and 76.3 (2)° out of the planes of the 4-methyl­phenyl and 2,4-dimethyl­phenyl rings, respectively. In the crystal, inter­molecular N—H⋯O hydrogen bonds link the mol­ecules into chains running along the *c* axis. The crystal studied was hemihedrally twinned with a twin law resulting from a twofold rotation about the *a* axis.

## Related literature

For the preparation, see: Gowda *et al.* (2003 ▶). For related structures, see: Bowes *et al.* (2003 ▶); Gowda *et al.* (2003 ▶, 2009**a* ▶,b* ▶, 2010 ▶).
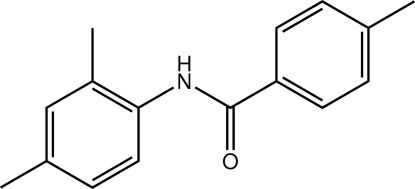


## Experimental

### 

#### Crystal data

C_16_H_17_NO
*M* *_r_* = 239.31
Monoclinic, 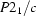

*a* = 22.4974 (17) Å
*b* = 6.6033 (2) Å
*c* = 9.2474 (6) Å
β = 100.209 (6)°
*V* = 1352.02 (14) Å^3^
*Z* = 4
Mo *K*α radiation
μ = 0.07 mm^−1^
*T* = 295 K
0.33 × 0.22 × 0.03 mm

#### Data collection

Oxford Diffraction Xcalibur, Ruby, Gemini diffractometer
Absorption correction: multi-scan (*CrysAlis PRO*; Oxford Diffraction, 2009 ▶) *T*_min_ = 0.983, *T*_max_ = 0.998
12970 measured reflections
3430 independent reflections
2107 reflections with *I* > 2σ(*I*)
*R*_int_ = 0.079

#### Refinement

*R*[*F* ^2^ > 2σ(*F* ^2^)] = 0.070
*wR*(*F* ^2^) = 0.192
*S* = 1.03
3430 reflections
167 parameters
1 restraint
H-atom parameters constrained
Δρ_max_ = 0.21 e Å^−3^
Δρ_min_ = −0.18 e Å^−3^

### 

Data collection: *CrysAlis PRO* (Oxford Diffraction, 2009 ▶); cell refinement: *CrysAlis PRO*; data reduction: *CrysAlis PRO*; program(s) used to solve structure: *SHELXS97* (Sheldrick, 2008 ▶); program(s) used to refine structure: *SHELXL97* (Sheldrick, 2008 ▶); molecular graphics: *ORTEP-3* (Farrugia, 1997 ▶) and *DIAMOND* (Brandenburg, 2002 ▶); software used to prepare material for publication: *SHELXL97*, *PLATON* (Spek, 2009 ▶) and *WinGX* (Farrugia, 1999 ▶).

## Supplementary Material

Crystal structure: contains datablocks I, global. DOI: 10.1107/S1600536810013413/tk2655sup1.cif

Structure factors: contains datablocks I. DOI: 10.1107/S1600536810013413/tk2655Isup2.hkl

Additional supplementary materials:  crystallographic information; 3D view; checkCIF report

## Figures and Tables

**Table 1:** Hydrogen-bond geometry (Å, °)

*D*—H⋯*A*	*D*—H	H⋯*A*	*D*⋯*A*	*D*—H⋯*A*
N1—H1n⋯O1^i^	0.86	2.07	2.884 (4)	159

Symmetry code: (i) 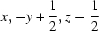.

**Fig. 1.**
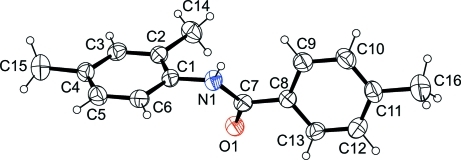
Molecular structure of (I) showing the atom labelling scheme. Displacement ellipsoids are drawn at the 30% probability level and H atoms are represented as small spheres of arbitrary radii.

**Fig. 2.**
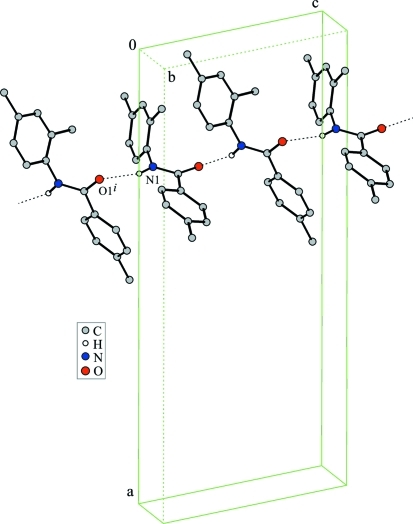
Part of crystal structure of (I) with hydrogen bonds shown as dashed lines. Symmetry code (i): x, -y+1/2, z-1/2. H atoms not involved in hydrogen bonding were omitted.

## supplementary crystallographic information

### Comment

As part of a study of the substituent effects on the crystal structures of benzanilides (Gowda *et al.*, 2003, 2009*a,b*, 2010), in the present work, the structure of *N*-(2,4-dimethylphenyl)4-methylbenzamide has been determined. In the structure, the N—H and C=O bonds are *anti* to each other (Fig. 1), similar to those observed in 4-methyl-*N*-(phenyl)benzamide (Gowda *et al.*, 2010), *N*-(2,6-dimethylphenyl)4-methylbenzamide (Gowda *et al.*, 2009*a*), *N*-(3,4-dimethylphenyl)4-methylbenzamide (Gowda *et al.*, 2009*b*) and the parent benzanilide (Bowes *et al.*, 2003). The benzene rings form a dihedral angle of 75.8 (1) °. The amide group is twisted by 28.1 (3) and 76.3 (2) ° out of the planes of the 4-methylphenyl and 2,4-dimethylphenyl rings, respectively. Intermolecular N–H···O hydrogen bonds (Table 1) link the molecules into chains running along the c axis of the crystal (Fig. 2).

### Experimental

The title compound was prepared according to the literature method (Gowda *et al.*, 2003). Plate-like colourless crystals were obtained from a slow evaporation of its ethanolic solution at room temperature.

### Refinement

Twinning was discovered, with two twin domains in a 1:1 ratio. and taken into account from the early stages of data collection. The twin law was determined as the matrix (-0.9998 0.0015 -0.8619/ -0.0001 -1.0000 -0.0003/ 0.0000 -0.0005 1.0001), which corresponds to a twofold rotation about the a axis. The non-diagonal matrix element of -0.8619 has a near-rational value of -6/7. Inspection of diffraction patterns and HKL files confirmed that reflections are overlapped mainly in the zones with *l* = 0 and *l* = 7. The twin scale factor was refined to a final value of 0.484 (2). All hydrogen atoms were positioned with idealized geometry using a riding model with C–H = 0.93 Å or 0.96 Å, and N–H = 0.86 Å, and with *U*_iso_(H) = 1.2*U*_eq_(C-aromatic, N) and 1.5*U*_eq_(*C*-methyl).

### Crystal data

**Table:**

C_16_H_17_NO	*F*(000) = 512
*M**_r_* = 239.31	*D*_x_ = 1.176 Mg m^−^^3^
Monoclinic, *P*2_1_/*c*	Mo *K*α radiation, λ = 0.71073 Å
Hall symbol: -P 2ybc	Cell parameters from 3071 reflections
*a* = 22.4974 (17) Å	θ = 1.8–29.6°
*b* = 6.6033 (2) Å	µ = 0.07 mm^−^^1^
*c* = 9.2474 (6) Å	*T* = 295 K
β = 100.209 (6)°	Plate, colourless
*V* = 1352.02 (14) Å^3^	0.33 × 0.22 × 0.03 mm
*Z* = 4	

### Data collection

**Table:**

Oxford Diffraction Xcalibur, Ruby, Gemini diffractometer	3430 independent reflections
graphite	2107 reflections with *I* > 2σ(*I*)
Detector resolution: 10.434 pixels mm^-1^	*R*_int_ = 0.079
ω scans	θ_max_ = 25.0°, θ_min_ = 2.8°
Absorption correction: multi-scan (*CrysAlis PRO*; Oxford Diffraction, 2009)	*h* = −26→26
*T*_min_ = 0.983, *T*_max_ = 0.998	*k* = −7→7
12970 measured reflections	*l* = −9→11

### Refinement

**Table:**

Refinement on *F*^2^	Primary atom site location: structure-invariant direct methods
Least-squares matrix: full	Secondary atom site location: difference Fourier map
*R*[*F*^2^ > 2σ(*F*^2^)] = 0.070	Hydrogen site location: inferred from neighbouring sites
*wR*(*F*^2^) = 0.192	H-atom parameters constrained
*S* = 1.03	*w* = 1/[σ^2^(*F*_o_^2^) + (0.0864*P*)^2^] where *P* = (*F*_o_^2^ + 2*F*_c_^2^)/3
3430 reflections	(Δ/σ)_max_ < 0.001
167 parameters	Δρ_max_ = 0.21 e Å^−^^3^
1 restraint	Δρ_min_ = −0.17 e Å^−^^3^

### Special details

**Table:**

Geometry. All esds (except the esd in the dihedral angle between two l.s. planes) are estimated using the full covariance matrix. The cell esds are taken into account individually in the estimation of esds in distances, angles and torsion angles; correlations between esds in cell parameters are only used when they are defined by crystal symmetry. An approximate (isotropic) treatment of cell esds is used for estimating esds involving l.s. planes.
Refinement. Refinement of *F*^2^ against ALL reflections. The weighted *R*-factor wR and goodness of fit *S* are based on *F*^2^, conventional *R*-factors *R* are based on *F*, with *F* set to zero for negative *F*^2^. The threshold expression of *F*^2^ > σ(*F*^2^) is used only for calculating *R*-factors(gt) etc. and is not relevant to the choice of reflections for refinement. *R*-factors based on *F*^2^ are statistically about twice as large as those based on *F*, and *R*- factors based on ALL data will be even larger.

### Fractional atomic coordinates and isotropic or equivalent isotropic displacement parameters (Å^2^)

**Table:**

	*x*	*y*	*z*	*U*_iso_*/*U*_eq_	
C1	0.19951 (16)	0.4402 (5)	−0.0180 (3)	0.0468 (9)	
C2	0.14244 (17)	0.3963 (5)	0.0076 (4)	0.0515 (9)	
C3	0.09535 (16)	0.5225 (6)	−0.0511 (4)	0.0567 (10)	
H3	0.0565	0.4918	−0.0368	0.068*	
C4	0.10472 (18)	0.6947 (6)	−0.1314 (4)	0.0600 (10)	
C5	0.16235 (19)	0.7348 (7)	−0.1540 (4)	0.0645 (11)	
H5	0.1695	0.8486	−0.2077	0.077*	
C6	0.20935 (17)	0.6099 (6)	−0.0986 (4)	0.0545 (10)	
H6	0.248	0.6389	−0.1151	0.065*	
C7	0.27622 (16)	0.2919 (5)	0.1727 (4)	0.0492 (9)	
C8	0.32400 (15)	0.1339 (5)	0.2078 (4)	0.0465 (9)	
C9	0.32238 (18)	−0.0436 (6)	0.1290 (4)	0.0679 (11)	
H9	0.2925	−0.0637	0.0469	0.081*	
C10	0.3653 (2)	−0.1920 (6)	0.1721 (5)	0.0699 (12)	
H10	0.363	−0.3127	0.1195	0.084*	
C11	0.41057 (18)	−0.1669 (6)	0.2888 (5)	0.0674 (12)	
C12	0.41121 (17)	0.0115 (7)	0.3662 (4)	0.0702 (11)	
H12	0.4417	0.0333	0.4467	0.084*	
C13	0.36811 (16)	0.1577 (6)	0.3279 (4)	0.0596 (11)	
H13	0.369	0.2745	0.3844	0.072*	
C14	0.13012 (19)	0.2119 (6)	0.0952 (5)	0.0733 (12)	
H14A	0.1481	0.2305	0.1965	0.11*	
H14B	0.1472	0.0942	0.0573	0.11*	
H14C	0.0873	0.1942	0.0872	0.11*	
C15	0.0514 (2)	0.8274 (7)	−0.1942 (5)	0.0921 (16)	
H15A	0.0653	0.9622	−0.2082	0.138*	
H15B	0.0233	0.8306	−0.1274	0.138*	
H15C	0.0319	0.7733	−0.2869	0.138*	
C16	0.4581 (2)	−0.3282 (7)	0.3361 (6)	0.1064 (17)	
H16A	0.4485	−0.4471	0.2769	0.16*	
H16B	0.459	−0.3615	0.4375	0.16*	
H16C	0.497	−0.2779	0.3239	0.16*	
N1	0.24865 (13)	0.3076 (4)	0.0336 (3)	0.0561 (8)	
H1N	0.2615	0.232	−0.0301	0.067*	
O1	0.26376 (11)	0.4035 (4)	0.2695 (3)	0.0578 (7)	

### Atomic displacement parameters (Å^2^)

**Table:**

	*U*^11^	*U*^22^	*U*^33^	*U*^12^	*U*^13^	*U*^23^
C1	0.056 (2)	0.056 (2)	0.0281 (19)	0.0081 (18)	0.0065 (16)	−0.0041 (17)
C2	0.069 (3)	0.055 (2)	0.029 (2)	0.0028 (19)	0.0041 (17)	−0.0007 (17)
C3	0.058 (2)	0.066 (3)	0.043 (2)	−0.005 (2)	0.0023 (17)	−0.005 (2)
C4	0.067 (3)	0.052 (3)	0.056 (3)	0.007 (2)	−0.0004 (19)	0.000 (2)
C5	0.080 (3)	0.056 (2)	0.057 (3)	−0.003 (2)	0.010 (2)	0.008 (2)
C6	0.063 (2)	0.056 (2)	0.044 (2)	−0.013 (2)	0.0079 (17)	−0.0021 (19)
C7	0.067 (2)	0.056 (2)	0.026 (2)	−0.0060 (17)	0.0125 (17)	−0.0031 (18)
C8	0.056 (2)	0.050 (2)	0.035 (2)	−0.0034 (15)	0.0135 (17)	0.0004 (17)
C9	0.087 (3)	0.059 (3)	0.053 (3)	0.005 (2)	0.002 (2)	−0.003 (2)
C10	0.090 (3)	0.051 (3)	0.068 (3)	0.008 (2)	0.011 (2)	−0.009 (2)
C11	0.068 (3)	0.049 (3)	0.087 (3)	0.006 (2)	0.016 (2)	0.013 (2)
C12	0.055 (2)	0.083 (3)	0.066 (3)	0.005 (2)	−0.0048 (19)	−0.001 (2)
C13	0.056 (2)	0.064 (3)	0.054 (2)	−0.003 (2)	−0.002 (2)	−0.012 (2)
C14	0.094 (3)	0.064 (3)	0.066 (3)	−0.003 (2)	0.025 (2)	0.014 (2)
C15	0.089 (3)	0.091 (4)	0.094 (4)	0.021 (3)	0.010 (3)	0.018 (3)
C16	0.099 (4)	0.093 (4)	0.124 (5)	0.021 (3)	0.011 (3)	0.013 (3)
N1	0.073 (2)	0.063 (2)	0.0321 (18)	0.0104 (17)	0.0092 (15)	0.0008 (15)
O1	0.0831 (18)	0.0566 (14)	0.0335 (13)	0.0090 (13)	0.0096 (12)	−0.0010 (13)

### Geometric parameters (Å, °)

**Table:**

C1—C2	1.377 (5)	C9—H9	0.93
C1—C6	1.385 (5)	C10—C11	1.357 (6)
C1—N1	1.424 (4)	C10—H10	0.93
C2—C3	1.381 (5)	C11—C12	1.377 (5)
C2—C14	1.514 (5)	C11—C16	1.518 (5)
C3—C4	1.394 (5)	C12—C13	1.370 (5)
C3—H3	0.93	C12—H12	0.93
C4—C5	1.375 (5)	C13—H13	0.93
C4—C15	1.515 (5)	C14—H14A	0.96
C5—C6	1.367 (5)	C14—H14B	0.96
C5—H5	0.93	C14—H14C	0.96
C6—H6	0.93	C15—H15A	0.96
C7—O1	1.230 (4)	C15—H15B	0.96
C7—N1	1.329 (4)	C15—H15C	0.96
C7—C8	1.492 (5)	C16—H16A	0.96
C8—C13	1.360 (5)	C16—H16B	0.96
C8—C9	1.377 (5)	C16—H16C	0.96
C9—C10	1.384 (5)	N1—H1N	0.86
			
C2—C1—C6	120.4 (3)	C10—C11—C12	117.1 (4)
C2—C1—N1	120.3 (3)	C10—C11—C16	122.4 (4)
C6—C1—N1	119.3 (3)	C12—C11—C16	120.6 (4)
C1—C2—C3	118.6 (3)	C13—C12—C11	121.8 (4)
C1—C2—C14	121.7 (3)	C13—C12—H12	119.1
C3—C2—C14	119.7 (4)	C11—C12—H12	119.1
C2—C3—C4	121.6 (4)	C8—C13—C12	120.7 (4)
C2—C3—H3	119.2	C8—C13—H13	119.7
C4—C3—H3	119.2	C12—C13—H13	119.7
C5—C4—C3	118.3 (3)	C2—C14—H14A	109.5
C5—C4—C15	122.2 (4)	C2—C14—H14B	109.5
C3—C4—C15	119.5 (4)	H14A—C14—H14B	109.5
C6—C5—C4	120.9 (4)	C2—C14—H14C	109.5
C6—C5—H5	119.5	H14A—C14—H14C	109.5
C4—C5—H5	119.5	H14B—C14—H14C	109.5
C5—C6—C1	120.2 (4)	C4—C15—H15A	109.5
C5—C6—H6	119.9	C4—C15—H15B	109.5
C1—C6—H6	119.9	H15A—C15—H15B	109.5
O1—C7—N1	122.0 (3)	C4—C15—H15C	109.5
O1—C7—C8	120.6 (3)	H15A—C15—H15C	109.5
N1—C7—C8	117.4 (3)	H15B—C15—H15C	109.5
C13—C8—C9	118.5 (3)	C11—C16—H16A	109.5
C13—C8—C7	119.3 (3)	C11—C16—H16B	109.5
C9—C8—C7	122.0 (3)	H16A—C16—H16B	109.5
C8—C9—C10	119.9 (4)	C11—C16—H16C	109.5
C8—C9—H9	120.1	H16A—C16—H16C	109.5
C10—C9—H9	120.1	H16B—C16—H16C	109.5
C11—C10—C9	122.0 (4)	C7—N1—C1	125.0 (3)
C11—C10—H10	119	C7—N1—H1N	117.5
C9—C10—H10	119	C1—N1—H1N	117.5
			
C6—C1—C2—C3	1.3 (5)	N1—C7—C8—C9	−29.8 (5)
N1—C1—C2—C3	−176.4 (3)	C13—C8—C9—C10	−0.4 (6)
C6—C1—C2—C14	179.9 (3)	C7—C8—C9—C10	−175.4 (4)
N1—C1—C2—C14	2.2 (5)	C8—C9—C10—C11	−1.8 (7)
C1—C2—C3—C4	−1.9 (5)	C9—C10—C11—C12	1.9 (6)
C14—C2—C3—C4	179.5 (3)	C9—C10—C11—C16	−179.4 (4)
C2—C3—C4—C5	1.4 (5)	C10—C11—C12—C13	0.1 (6)
C2—C3—C4—C15	179.7 (4)	C16—C11—C12—C13	−178.6 (4)
C3—C4—C5—C6	−0.3 (6)	C9—C8—C13—C12	2.3 (6)
C15—C4—C5—C6	−178.6 (4)	C7—C8—C13—C12	177.4 (3)
C4—C5—C6—C1	−0.2 (6)	C11—C12—C13—C8	−2.2 (6)
C2—C1—C6—C5	−0.3 (5)	O1—C7—N1—C1	−5.1 (6)
N1—C1—C6—C5	177.5 (3)	C8—C7—N1—C1	175.7 (3)
O1—C7—C8—C13	−24.1 (5)	C2—C1—N1—C7	−75.0 (4)
N1—C7—C8—C13	155.2 (3)	C6—C1—N1—C7	107.3 (4)
O1—C7—C8—C9	150.9 (4)		

### Hydrogen-bond geometry (Å, °)

**Table:**

*D*—H···*A*	*D*—H	H···*A*	*D*···*A*	*D*—H···*A*
N1—H1n···O1^i^	0.86	2.07	2.884 (4)	159

Symmetry codes: (i) *x*, −*y*+1/2, *z*−1/2.
